# Genetic diversity of US Rambouillet in the National Sheep Improvement Program compared to other sheep breeds

**DOI:** 10.1093/jhered/esaf079

**Published:** 2025-10-03

**Authors:** Emily J Schulz, Sara M Nilson, Tom W Murphy, Brenda M Murdoch, Luiz F Brito, Ronald M Lewis, Jessica L Petersen

**Affiliations:** Department of Animal Science, University of Nebraska–Lincoln, 3940 Fair St, Lincoln, NE, 68583-0908, United States; Department of Animal Science, University of Nebraska–Lincoln, 3940 Fair St, Lincoln, NE, 68583-0908, United States; USDA, ARS, Livestock Bio-Systems Research Unit, Roman L. Hruska U.S. Meat Animal Research Center, State Spur 18D, Clay Center, NE, 68933, United States; Department of Animal, Veterinary and Food Science, University of Idaho, 875 Perimeter Dr, Moscow, ID, 83844-2330, United States; Department of Animal Sciences, Purdue University, 270 S Russell St, West Lafayette, IN, 47907, United States; Department of Animal Science, University of Nebraska–Lincoln, 3940 Fair St, Lincoln, NE, 68583-0908, United States; Department of Animal Science, University of Nebraska–Lincoln, 3940 Fair St, Lincoln, NE, 68583-0908, United States

**Keywords:** inbreeding, *Ovis aries*, population management

## Abstract

Breed management and genomic evaluation rely on understanding population structure and genetic diversity. The primary objective of this study was to evaluate genetic diversity in Rambouillet enrolled in the National Sheep Improvement Program (NSIP) in comparison to other US and international sheep breeds. We considered genotypes of 667 NSIP Rambouillet from a 50 K single nucleotide polymorphism (SNP) array and 600 K SNP genotypes on 64 each of NSIP Rambouillet, Suffolk, and Katahdin sheep. Pedigree analyses were also conducted on the NSIP Rambouillet. International comparisons incorporated 50 K SNP data from nine international breeds. After genomic quality control measures to reduce bias in analyses, the NSIP Rambouillet had the greatest diversity among the three NSIP breeds (expected heterozygosity: 0.404; average individual inbreeding: 9.94%). Conversely, the NSIP Rambouillet had the lowest genetic diversity when compared to the international breeds. Based on principal component analyses, NSIP Rambouillet were divergent from the international populations except for evidence of connectivity between the NSIP and European Rambouillet. Population structure within the NSIP Rambouillet, demonstrated by cluster analysis and a significant loss of heterozygosity (F_IS_) was driven primarily by one flock. Using complete pedigrees of the NSIP population, effective population size, effective number of founders, and average generation interval was 87 to 218, 95, and 3.4 years, respectively. This pedigree and genomic assessment of genetic diversity provides the basis for genomic selection and monitoring of the NSIP Rambouillet.

## Introduction

Developed from Spanish Merino sheep in France and Germany, Rambouillet sheep have been a cornerstone breed in Western range flocks since their introduction into the United States in the 1800s (Dickinson and Lush 1933). The Rambouillet breed is known for its fine-diameter wool and heavy fleece production, the quality of which has allowed this breed to dominate the US wool industry. Still, the Rambouillet is considered a dual-purpose breed valued for its contributions to lamb production. Selection pressure over the past 50 years has resulted in increased average daily gain and mature size of Rambouillet sheep (Burton et al. 2015). The Rambouillet breed is also characterized by its relatively long breeding season and hardiness across climatic regions and is therefore often used in crossbreeding programs and in the development of composite breeds. For instance, at the US Sheep Experimentation Station, the Rambouillet was used in the creation of the Targhee, Columbia, and Polypay breeds (Ercanbrack and Knight 1981; Ercanbrack and Knight 1998; Rasali et al. 2006). Similarly, in India, the Rambouillet was used heavily to improve local breeds and has been credited for significant gains in both wool and meat qualities (Hamadani et al. 2019).

The US sheep industry has witnessed a drastic decline in population size, dropping from 49 million animals in the 1940s to ~5 million animals in 2023 (USDA, 2023; Stillman 1990). The reasons for this reduction in the number of sheep are multi-faceted (Thorne et al. 2021), but raises concern regarding the genetic diversity of the species, particularly when considering individual breeds. In recent years, trends within the sheep industry have led to a shift in the focus of the US market from wool to meat production (Gootwine 2020). For instance, due to recent growth in non-traditional lamb marketing channels, an exponential increase in demand for lamb is forecasted (Shiflett et al. 2010; Williams et al. 2011), which may stimulate growth in the US sheep inventory. Given the historical decline in the US sheep population, any resurgence in the US sheep inventory would thus arise from a smaller genetic base. Although inbreeding, and thus the loss of genetic diversity can help refine populations, the accumulation of inbreeding, particularly over short periods of time, can result in a reduction in animal performance (Leroy 2014; Doekes et al. 2021). Therefore, a genetic evaluation of the breed is not only necessary for determining the current state of diversity of the Rambouillet but has crucial implications on the sustainability of the US sheep industry.

The National Sheep Improvement Program (NSIP) was first established in the 1980s and remains the only national genetic evaluation of US sheep breeds (Wilson and Morrical 1991; Notter 1998). Rambouillet accounted for 11.4% of the over 130,000 lambs evaluated in the NSIP from 2020–2024, third behind Katahdin (33.3%) and Polypay (24.4%; www.nsip.org). With genotyping becoming more affordable, many livestock breeds have begun incorporating genomic information into their genetic evaluation services to determine parentage, estimate relationships, track economically important genetic markers, and perform selective breeding. The Katahdin was the first NSIP breed to have genomically-enhanced estimated breeding values available (McMillan et al. 2022) and foundation work has begun in other breeds including Rambouillet (Araujo et al. 2023). Additional examples of recent genetic studies within Rambouillet include genome-wide association studies (Becker et al. 2022) and genetic parameter estimation (Gunes et al. 2024). An understanding of genetic diversity in US Rambouillet would complement these studies by providing a basis for monitoring changes in diversity over time, which is necessary for establishing a sustainable breeding program.

Genomic diversity of US Rambouillet sheep has been previously assessed in conjunction with other US sheep breeds using microsatellite data (Blackburn et al. 2011) and single nucleotide polymorphism (SNP) genotypes (Zhang et al. 2013; Becker et al. 2024). However, a comprehensive evaluation of the NSIP Rambouillet alone and in comparison to other breeds is lacking. Therefore, the primary aim of this study was to characterize the current level of genetic diversity among NSIP Rambouillet sheep using pedigree and genomic data. A further aim was to compare the genetic diversity of the NSIP Rambouillet population to two other prominent NSIP breeds and to international breeds genetically similar to the Rambouillet.

## Methods

No ethical review and approval were needed for this study as all datasets used were provided by commercial breeding operations.

### Data

#### Genomic data

DNA samples were collected by NSIP producers on a Neogen (Lincoln, NE, USA) blood card after jugular venipuncture. Animal ID and pedigree (as available) were recorded. Samples representing 64 individuals each of Rambouillet (44 male, 20 female), Suffolk (63 male, 1 female), and Katahdin (all male), all recorded with NSIP, were sent to Neogen (Lincoln, NE, USA) for genotyping on the Illumina Ovine HD 600 K SNP array (606,006 loci). An additional batch of 676 NSIP Rambouillet representing 297 rams and 379 ewes were genotyped at Neogen using the GeneSeek Genomic Profiler Ovine 50 K array (51,867 SNPs). The Rambouillet sheep genotyped represented nine flocks and sampled animals were born between 2009 and 2017. Although specific flock identification is confidential, the nine flocks represented six states: Kansas, Montana (2), Ohio, South Dakota (3), Texas, and Utah. Based on pedigree, the genotyped sheep were offspring of 43 sires and 557 dams.

For additional population comparisons, 50 K Illumina SNP data were obtained from Kijas et al. (2012); data from nine populations, either wool breeds or breeds genetically similar to the Rambouillet, were chosen for comparison to the NSIP Rambouillet (‘international data’; Table 1).

**Table 1:** Sample size of breeds included in the comparison of international sheep. Except for the NSIP Rambouillet, the other samples originated in Southwest Europe (data from Kijas et al. 2012). ^*^One NSIP Rambouillet was removed as it was the only representative of its flock.

Breed	Initial N	N after Removing First-Degree Relationships
Australian Industry Merino	88	77
Australian Merino	50	42
Australian Poll Merino	98	77
Chinese Merino	23	23
Churra	120	100
Merinolandschaf	24	21
Ojalada	24	24
NSIP Rambouillet	740	500^*^
European Rambouillet	102	99
Rasa Aragonesa	22	20

#### Pedigree data

Pedigree information on the entire NSIP Rambouillet population from 1985 through 2020 was obtained. Animals with missing parents and no recorded progeny were removed from analyses (*n* = 178). The final pedigree included 15,543 male and 16,890 female animals, which were offspring of 755 sires and 7,352 dams. The sire or dam was unknown for 4.1% and 2.4% of these animals, respectively, with both unknown for 6.3%. Twenty-five Rambouillet flocks were represented in the complete pedigree.

### Genotyping and quality control

To combine data from SNP arrays, the position of each SNP was remapped to the Rambouillet ver2.0 reference genome (Davenport et al. 2022) using the NCBI Genome Remapping Service. Loci were also manually checked to recover positions of markers that did not have coordinates noted (where possible) as well as to identify duplicate markers. Updated map positions were included in PLINK files allowing for the combining of data from both arrays. 619,450 autosomal loci present in the merged data set, with an overall genotyping rate of 15% reflecting the fact that most animals were not genotyped on the 50 K array.

PLINK ver 2.0 (Purcell et al. 2007) was used to remove loci with a genotyping rate less than 95% (effectively removing SNPs not shared across arrays), those with a minor allele frequency less than 0.02 across all samples, and non-autosomal or unmapped SNP. Individual sheep were removed if they had greater than 5% missing genotype data. To reduce the potential impact of linkage disequilibrium (LD), PLINK was used to randomly select a SNP every 50,000 base pairs (−bp-space); this method was chosen based upon known decay of LD in sheep (Kijas*,* et al. 2014). Reducing LD in this manner also serves to avoid bias due to the overrepresentation of genotypes within flocks or breeds as is possible if empirical data are instead used to identify regions of LD.

Employing methods from Manichaikul et al. (2010) in PLINK, first-degree relationships were removed from among each sample using the flag –king-cutoff with a threshold of 0.177 to avoid possible bias due to the inclusion of large family groups. Given this method, a KING kinship coefficient of ~ 0.354 (range from 0.25 to 0.5) is expected for identical samples and monozygotic twins and therefore a mean of ~ 0.177 is expected to distinguish first degree relationships. Katahdin, Suffolk, and Rambouillet NSIP flocks were then represented by 49, 52, and 501 individuals, respectively. As one NSIP Rambouillet flock was represented by only one individual, data from that sheep were removed for flock comparisons (final N for NSIP Rambouillet = 500) (Table 1).

For expected heterozygosity, population-level inbreeding (F_IS_), and differentiation (F_ST_) analyses, the number of individuals evaluated within each population sample was additionally thinned by random selection using the –thin-indiv-count flag in PLINK to maintain similar sample size among the different breeds. This thinning was performed in three independent iterations. For comparisons among the three NSIP breeds (Rambouillet, Katahdin, Suffolk), each was thinned to include 49 individuals. In comparisons of the NSIP Rambouillet with the international dataset, samples were thinned to include 20 to 24 per breed.

Genotype-based inbreeding coefficients were calculated for all genotyped individuals (without removal of relationships) after first calculating expected heterozygosity (H_e_) from the LD-pruned and thinned datasets to avoid bias due to LD or differences in sample size per breed. To do so the –geno 0.0 flag, along with –bp-space 50,000 was employed in PLINK to create a consistent marker set for each of the three thinned datasets within each subset of data (NSIP and international). The –het flag in PLINK was then used to determine mean H_e_ for each of the thinned files. The expected heterozygosity was averaged among the three thinned files for independent analyses of the NSIP and international subsets. The observed homozygosity (H_o_) of all samples (regardless of relationship), calculated in PLINK, and the mean expected heterozygosity were used to calculate individual estimates of genomic inbreeding as $F=\frac{H_e-{H}_o}{H_e}$.

### Pedigree analyses

The number of ancestors, effective ancestors and founders, and count of ancestors explaining 50% of the genetic diversity in the population were estimated by ENDOG v4.8 (Gutierrez and Goyache 2005) for the entire pedigree and for only those sheep included in the genomic analyses.

Pedigree-based estimates of effective population size (N_e_) were calculated in ENDOG v4.8, with the individual increase in inbreeding coefficients adjusted by maximum, complete, and equivalent generations (Gutierrez and Goyache 2005). In addition, N_e_ was estimated from the regression and log regression of (1-F_t_) on birth date (where F_t_ is inbreeding at time t) adjusted for generation interval (Perezenciso 1995).

The pedigree-based inbreeding coefficient for each sheep included in the genomic analysis was calculated in ENDOG v4.8 using all available pedigree information. The correlations between the pedigree- and genomic-based estimates were determined.

The generation interval was estimated for the NSIP Rambouillet pedigree along the four pathways of sire-son, sire-daughter, dam-son, and dam-daughter. Generation interval was defined two ways: as the average age of parents at the birth of their progeny kept for reproduction (GI_kept_), or as the average age of parents at the birth of their progeny regardless of whether the lamb was kept for reproduction (GI_all_).

### SNP-based population diversity

Utilizing the LD-pruned marker set, as an estimate of diversity, the expected heterozygosity for each breed sample was calculated in Arlequin 3.11 (Excoffier and Lischer 2010). PGD Spider (Lischer and Excoffier 2012) was utilized to convert input files from PLINK format to the format necessary for analyses.

F_IS_ values were calculated after the same pruning criteria in Genetix (Belkhir et al. 1996-2004) with significance determined by 500 permutations. The calculation was conducted in triplicate using the three random subsets of the data.

Historical effective population size (N_e_) for 13 generations prior to the most recent samples was estimated with SNeP v1.11 (Barbato et al. 2015). Two sets of data were analyzed: 1) all NSIP Rambouillet considering 32,910 markers shared across both SNP genotyping arrays, pruned for kinship as described above (*n* = 500), and 2) all NSIP Rambouillet genotyped on the 50 K array including 40,161 markers, also pruned for kinship (*n* = 466). Due to differences in SNP density between arrays, the number of pairwise comparisons required for a bin (50 kb) to be included in calculating N_e_ was set to 75 and 150 for datasets 1 and 2, respectively. The estimate of most recent N_e_ was predicted by fitting a linear model to the historical estimates.

### Relationships among samples

Utilizing all sheep in each sample set (NSIP and international; genotyped on either SNP array), principal component analysis (PCA) was conducted in PLINK to visualize variation among flocks and breeds and repeated after randomly sampling flocks (in the case of the NSIP Rambouillet) or breeds (in the case of the International comparison) to account for unequal sample sizes in the full data set. The final number of SNPs included in each analysis was determined by maf and genotyping thresholds as described and thus varied slightly depending upon the individuals included. Pairwise F_ST_ among samples was calculated using Reynold’s distance in Arlequin (Excoffier and Lischer 2010); significance was determined using 1,000 permutations.

Cluster analyses of the NSIP Rambouillet alone and with the international dataset were conducted in fastSTRUCTURE (Raj et al. 2014), using the datasets thinned to remove first-degree relationships and high LD. K values from 1 to 15 were considered and the most likely number of clusters was evaluated using the method of Puechmaille (2016) implemented in StructureSelector (Li and Liu 2018).

**Fig. 1:**
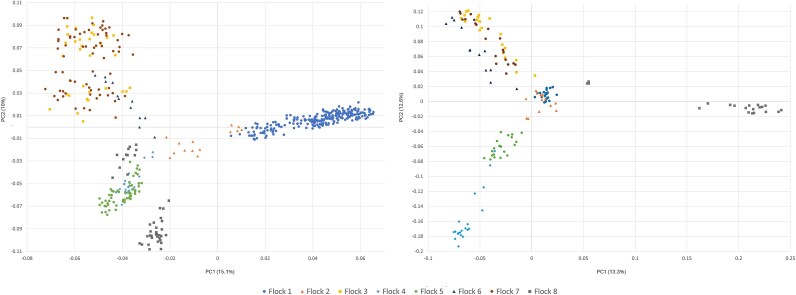
(left) Principal component (PC)1 and PC2 for individuals within the NSIP Rambouillet population grouped by flock. Analysis represents 8 flocks within the United States after thinning the sample to remove first-degree relationships. (right) PC1 vs PC2 for the NSIP Rambouillet after thinning flocks in the prior data set that had more than 20 individuals to *n* = 20. Both analyses included 21,098 SNPs.

**Fig. 2:**
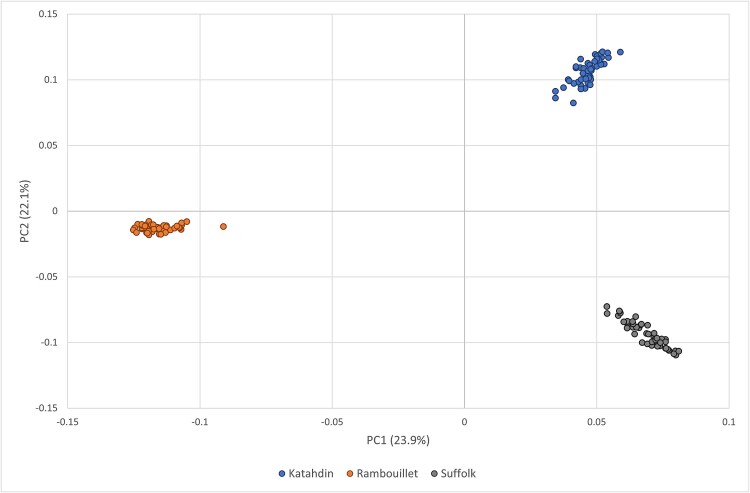
Principal component analysis (PC 1 vs PC2) of the NSIP Rambouillet, Katahdin and the Suffolk samples after subsampling to equalize sample size (*n* = 49 each). The analysis included 22,169 SNPs.

## Results

### Genomic analyses of NSIP breed samples

After removing close relationships and the single individual representing one flock as described, the full NSIP Rambouillet genomic data set included 500 sheep. These sheep represented 8 flocks with 12 (Flock 6) to 247 (Flock 1) sheep per flock (mean: 62.5). From the full dataset, the first two PCs in the analysis of the 8 NSIP Rambouillet flocks (21,098 SNPs) identified clustering of Flocks 3 and 7 and of Flocks 4 and 5, all split from the Flock 1 on PC1 (Fig. 1). Flock 2 was positioned intermediate between Flock 1 and the others along PC1 with Flock 6 intermediate between Flock 2 and Flocks 3 and 7. Flock 8 was split across Flocks 4 and 5. The third PC served only to distance Flock 8 from the others on the left side of PC1 (Supplementary Fig. 1). The proportion of the total variance accounted for by the first principal component was 15.1%, with 10.0% and 9.3% explained by PC2 and PC3, respectively. When subsampling the flocks to include a maximum of 20 individuals each, Flocks 1 and 2 overlapped one another with Flock 8 positioned as the most distinct on PC1. The relationships among Flocks 3, 5, and 7 were maintained while Flocks 4 and 5 separated along PC1.

After subsampling to equalize sample size per breed (*n* = 49), principal component analysis of NSIP Rambouillet, Suffolk, and Katahdin demonstrated clear distinction between the Rambouillet and the others on PC1 (Fig. 2), which explained 23.9% of the variation. The Suffolk and Katahdin showed separation along PC2, which explained a similar proportion (22.1%) of the variation in the data. If all 500 NSIP Rambouillet were included, however, substructure within the Rambouillet became evident across PC2 (Supplementary Fig. 2). Pairwise F_ST_ of the NSIP samples demonstrated similar levels of divergence among the three flocks with all F_ST_ values significantly greater than zero (*P* < 0.001; Table 2).

**Fig. 3:**
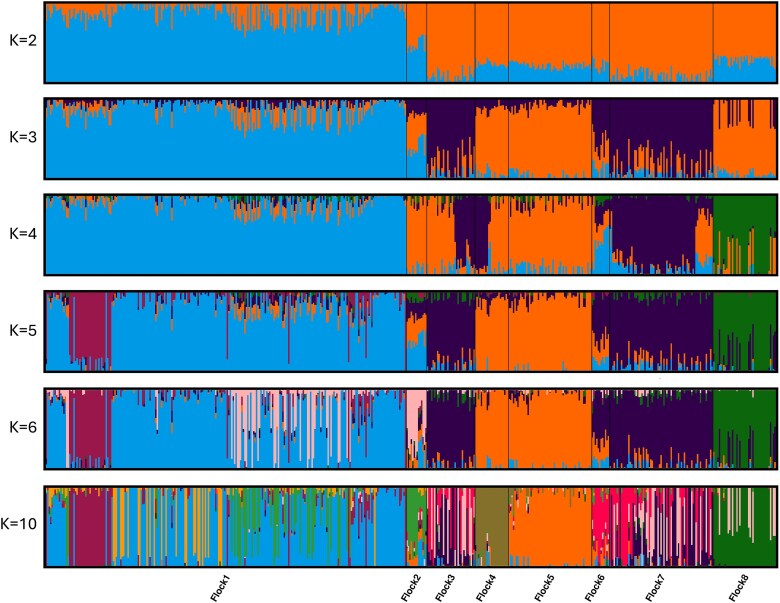
Cluster plots of the NSIP Rambouillet assuming K = 2, 3, 4, 5, 6, and 10 (*n* = 500). The analysis included 20,743 SNPs.

**Table 2:** Pairwise F_ST_ values comparing NSIP samples of Katahdin, Rambouillet, and Suffolk sheep. All comparisons are significantly greater than zero (*P* < 0.001).

NSIP breed	Katahdin	Rambouillet
Katahdin	0	
Rambouillet	0.135	0
Suffolk	0.132	0.133

Expected heterozygosity of the three NSIP breeds was greatest in the Rambouillet (0.402), with the Suffolk intermediate (0.393), and Katahdin the lowest (0.388). The Katahdin however, was the only sample with F_IS_ (0.001) not significantly greater than zero. The F_IS_ of the Rambouillet (0.023) and Suffolk (0.035) differed from zero (*P* < 0.001) in all three replicates of data analysis. Among these NSIP groups, mean individual inbreeding was lowest in Rambouillet (0.092), intermediate in Katahdin (0.106), and greatest in Suffolk (0.130). The NSIP Rambouillet contained individuals with both the lowest (−0.020) and highest (0.346) individual genomic inbreeding coefficients (F) of the three NSIP breeds (Table 3). The SNP-based estimates of effective population size were 383.2 and 401 when only data from the 50 K array was used and when data across arrays was used, respectively (Table 4).

**Table 3:** The minimum, maximum, and average individual inbreeding coefficients calculated for each of the US breeds determined from 17,266 SNP.

Individual Inbreeding Coefficients (F)			
NSIP breed	Minimum	Maximum	Average
Katahdin	0.044	0.213	0.106
Rambouillet	−0.020	0.346	0.092
Suffolk	0.051	0.294	0.130

**Fig. 4:**
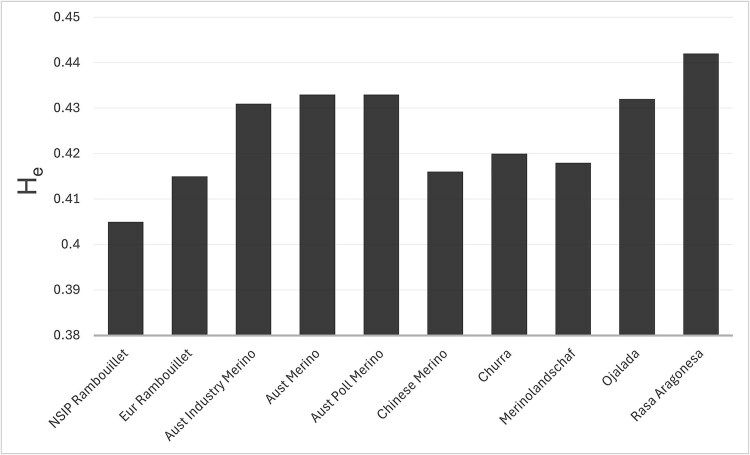
Expected heterozygosity for the NSIP Rambouillet compared to other wool breeds. Data were calculated with 16,793 SNPs after large samples were subsampled (three replicates) to remove bias due to unequal sample size. Note the y-axis scale.

Based on cluster analysis of the NSIP Rambouillet, the Puechmaille method suggested the optimal number of clusters was 6 (threshold = 0.5; Fig. 3). At K = 6, Flocks 3 and 7 primarily assigned to the same cluster as Flocks 4 and 5. At K = 6, structure within Flock 1 became evident. Flock 2 assigned primarily to one cluster, shared with some individuals of Flock 1, but also showed admixture from the other clusters. Flock 6 was most similar to Flock 7 but with also shared ancestry with the cluster to which Flocks 4 and 5 assigned. Increased values of K served to increase substructure within the flocks, initially observed in Flocks 3 and 6 but eventually in Flocks 1 and 8 as well. At K = 10, Flock 4 was primarily assigned to a unique cluster, sharing ancestry with some individuals of Flock 1.

Pairwise F_ST_ among the NSIP Rambouillet flocks demonstrated significant divergence between all flocks except for Flocks 3 and 7 (Supplementary Table 1).

### Pedigree-based analyses of NSIP Rambouillet

The complete NSIP Rambouillet pedigree contained 32,433 individuals. For the entire pedigree, completeness was 90.4% within one generation (parents) and did not decay below 50% until four generations (great-great-grandparents) prior to the proband individuals. For those sheep from which genotypes were available, the pedigree completeness was 99% for the parental generation. There were 2,342 ancestors for the complete pedigree, with 109 effective ancestors and 95 effective founders. When limited to genotyped animals, there were a total of 398 ancestors with 55 effective ancestors and 78 effective founders. Tracing pedigree information of all NSIP Rambouillet genotyped sheep found that ancestors of the pedigree were largely reported as Rambouillet (75.1%), unknown or unreported ancestry (23.7%), or Merino (1.2%). Merino ancestors were only present in the pedigree of Flock 1, which also had the greatest proportion of ancestors classified as unknown (44.5%). The number of ancestors explaining 50% of the genetic diversity were 51 and 19 for the complete pedigree and the genotyped animals, respectively. Generation interval considering all individuals (GI_all_) ranged from 2.9 (father-daughter) to 4.2 (mother-son) years, averaging 3.4 years; the average was also 3.4 years when calculated using only those sheep which had progeny kept for reproduction (GI_kept_). Utilizing the entire NSIP Rambouillet pedigree, effective population size ranged from 87 to 218 (Table 4). Considering only those sheep genotyped, the estimated effective population size determined by regression or log-regression on birthdate averaged 157.4.

Pedigree-based inbreeding coefficients for the genotyped samples ranged from 0 to 0.25; 417 (57%) of the genotyped sheep (excluding those without known parents) had an inbreeding coefficient of 0 based upon pedigree. Inbreeding estimates were positively correlated with the number of complete generations available in the pedigree (r = 0.39). Although the mean individual inbreeding coefficient increased over time, the rate of increase did not differ across years for which pedigree data were available (*P* = 0.67). The correlation between pedigree and genotype-based inbreeding estimates of the genotyped sheep was 0.34 (Supplemental Fig. 3). If only sheep with 2 or more complete generations of pedigree were considered (*n* = 314), the correlation increased to 0.44. For sheep with 3 or more complete generations of pedigree, the correlation increased further to 0.64 (*n* = 236). If sheep with F of zero, as determined by pedigree, were removed, the correlation between pedigree and genotype estimates of inbreeding was 0.72, regardless of pedigree depth.

**Table 4:** Estimates of effective population size (N_e_) based on entire NSIP Rambouillet pedigree, from pedigree data of only the sheep genotyped, and from genomic data of the sheep genotyped.

	Method	N_e_
Pedigree (all NSIP)	Increase in inbreeding (maximum generation)	218.3
	Increase in inbreeding (equivalent generation)	87.1
	Regression on birthdate	181.0
	Log-regression on birthdate	172.2
Pedigree (genotyped sheep)	Regression on birthdate	139.8
	Log-regression on birthdate	174.9
Genomic	Data set 1 (both SNP arrays)	401.0
	Data set 2 (only 50 K data)	383.2

### Comparison to international sheep breeds

The diversity of each sample was estimated by H_e_ using 16,793 markers across three randomly sampled iterations of the data (227 sheep per dataset). The Rasa Aragonesa had the highest expected heterozygosity (0.442), followed by the Australian Merino (0.443), the Australian Poll Merino (0.433), and the Australian Industry Merino (0.431; Fig. 4). The NSIP Rambouillet demonstrated the lowest H_e_ (0.405) of these samples of wool breeds. The estimates from each of the three subsets of the data (thinned to remove bias due to sample size) were similar, varying 0.1% on average (0.8% maximum) from the mean estimate per sample.

The Chinese Merino had the lowest F_IS_ (−0.014) which, along with that of the Merino Landschaf and Ojalada, was not significantly different from zero. The F_IS_ values of all other samples were significantly greater than zero including the NSIP Rambouillet, which had the greatest F_IS_ (0.038; *P* < 0.001), like that of the European Rambouillet sample (0.037; *P* < 0.001) (Supplementary Table 2).

When comparing individual genomic inbreeding coefficients, the NSIP Rambouillet had the greatest average inbreeding (11.8%) followed by the European Rambouillet (9.7%; Table 5). The European Rambouillet sample contained the individual with the lowest inbreeding coefficient (−4.7%) while the individual with the highest inbreeding value (37.9%) was from the NSIP Rambouillet sample. Mean genomic inbreeding of the other international samples ranged from 0.1% (Rasa Aragonesa) to 6.1% (Churra).

**Table 5:** Genotype-based individual inbreeding estimates for the NSIP Rambouillet and the 9 international breeds.

	Minimum	Maximum	Average
Australian Industry Merino	−0.011	0.121	0.039
Australian Merino	0.002	0.219	0.048
Australian Poll Merino	−0.010	0.132	0.027
Chinese Merino	−0.015	0.134	0.045
Churra	−0.005	0.277	0.061
Merinolandschaf	0.021	0.104	0.055
Ojalada	−0.011	0.072	0.021
Rasa Aragonesa	−0.021	0.063	0.001
NSIP Rambouillet	0.018	0.379	0.118
European Rambouillet	−0.047	0.323	0.097

**Fig. 5:**
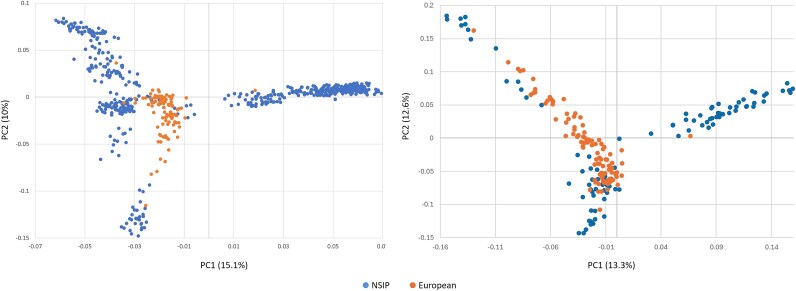
Principal component analysis (PC1 vs PC2) for (left) all Rambouillet (NSIP and European; *n* = 602) and (right) using an equal sample of NSIP and European Rambouillet (*n* = 102 each). Both analyses were conducted with 21,062 SNPs.

Principal component analysis was conducted using the LD pruned marker set to compare the entirety of the NSIP Rambouillet sample to the individuals in the European Rambouillet sample (Fig. 5). When all 500 NSIP Rambouillet were included, the sample formed two clusters divided across PC1 with the European sample falling intermediate to the two NSIP groupings; 97.6% of the sheep on the right side of the plot represented Flock 1. Variance explained by the first 2 PCs was 15.1% and 10.1%, respectively. When the dataset was thinned to make sample sizes more similar (*n* = 102 each in the NSIP and European samples), the European Rambouillet remained intermediate between Flock 1 and the other flocks. However, overlap with the non-Flock 1 sheep became more evident.

**Fig. 6:**
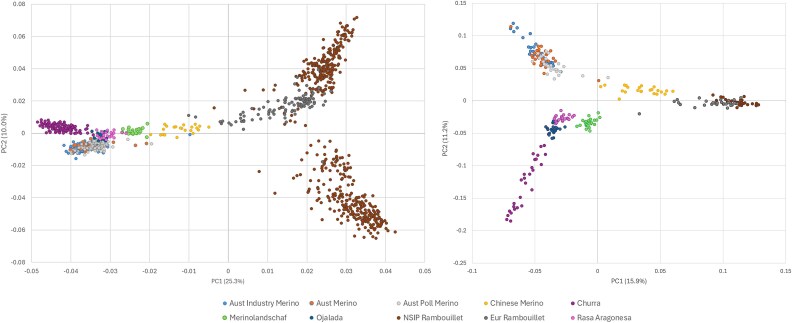
Principal component plot of 9 international wool breeds and the NSIP Rambouillet including (left) all samples (*n* = 1052 sheep and 16,793 SNPs) and (right) after randomly sampling *n* = 30 individuals per breed (16,791 SNPs).

Based on PCA comparing the nine international population samples and all NSIP Rambouillet, the NSIP Rambouillet diverged across PC2 (Fig. 6). As observed in other analyses, the European Rambouillet population was intermediate to the NSIP Rambouillet. The Chinese Merino was most proximal to Rambouillet on PC1, which accounted for 25.3% of the variation observed; PC2 accounted for 10.0% of the variation. The Australian Industry Merino, Australian Merino, Australian Poll Merino, Ojalada, and Rasa Aragonesa samples overlapped. The third PC (8.3%) regrouped the NSIP Rambouillet into a single cluster, further dividing the Churra from the other samples (Supplementary Fig. 4). Subsampling each to include a maximum of 30 individuals per breed preserved the relationship between the NSIP and European Rambouillet with the Chinese Merino while further separating the Australian Merinos from the other wool breeds on PC2.

From the analyses of F_ST_, the Churra and NSIP Rambouillet samples had the greatest differentiation among all comparisons (Table 6), which was also suggested by PCA. The average F_ST_ between the Australian Industry Merino and Australian Merino populations was the only comparison (F_ST_ = 0.002) not statistically different from zero (*P* > 0.05).

**Table 6:** Mean pairwise F_ST_ values calculated from three subsets of data including the international breeds and the NSIP Rambouillet. All F_ST_ values demonstrate significant differentiation except for the Australian industry merino and the Australian merino, which did not differ from one another in 1 of the 3 replicates (*P* = 0.39).

	Aust Industry Merino	Aust Merino	Aust Poll Merino	Chinese Merino	Churra	Merino-landschaf	Ojalada	NSIP Rambouillet	Eur Rambouillet
Aust Merino	0.002								
Aust Poll Merino	0.009	0.011							
Chinese Merino	0.049	0.047	0.046						
Churra	0.052	0.049	0.050	0.069					
Merinolandschaf	0.053	0.051	0.051	0.063	0.063				
Ojalada	0.038	0.036	0.037	0.054	0.037	0.049			
NSIP Rambouillet	0.067	0.064	0.063	0.058	0.084	0.075	0.069		
Eur Rambouillet	0.053	0.050	0.050	0.045	0.068	0.060	0.054	0.015	
Rasa Aragonesa	0.025	0.024	0.024	0.042	0.029	0.037	0.015	0.057	0.042

**Fig. 7:**
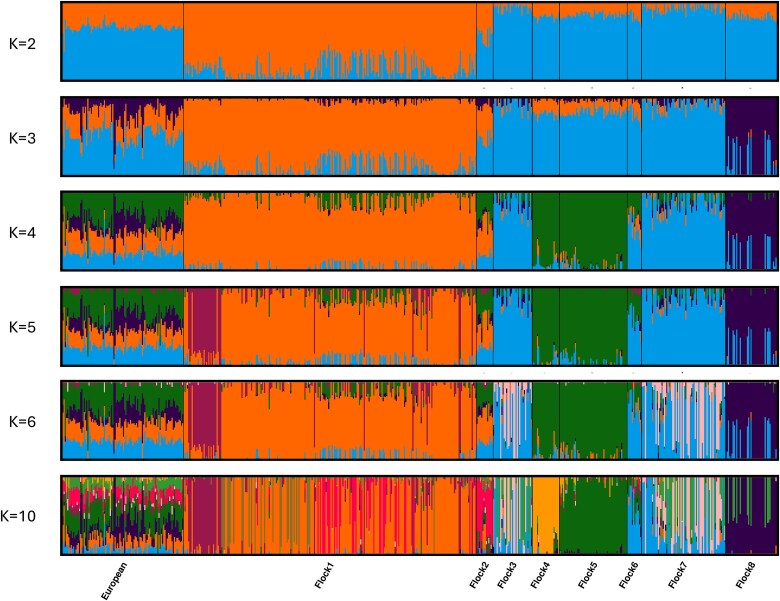
Cluster plot (K = 2, 3, 4, 5, 6, and 10) of the NSIP and European Rambouillet samples (*n* = 602) utilizing 21,062 SNPs.

Based on cluster analysis of the NSIP Rambouillet and the European Rambouillet together, the data suggest the ideal number of clusters was 6 with the results mirroring that of the cluster analysis conducted on only NSIP sheep (Fig. 7). Even at K = 2, the individuals did not cluster based upon their geographic origin (United States vs Europe) but instead the European samples showed shared ancestry from all clusters evident in the NSIP samples. At K = 6, the European sample shared nearly equal proportions of ancestry with the majority of Flock 1, the cluster representing Flocks 3 and 7, the cluster representing Flocks 4 and 5, and that of Flock 8. Even at K = 10, the ancestry of the European sample was parsed among the NSIP clusters.

## Discussion

The goal of this study was to use genotype and pedigree data to evaluate the genetic diversity of Rambouillet sheep participating in the NSIP. Using pedigree, there was no indication of an increase in the rate of inbreeding over time. However, also based on pedigree, there were a relatively small number of founders responsible for the current population. Nevertheless, genomic data analyses suggested that NSIP Rambouillet had greater diversity than two other NSIP breeds studied, although lesser diversity than the international breeds that were considered. Population substructure was evident within NSIP Rambouillet, primarily driven by divergence of the largest flock (Flock 1) but also evident in more subtle substructure among other flocks. This population structure is likely responsible for the significant F_IS_ observed for the NSIP Rambouillet, like that reported in NSIP Suffolk (Wilson et al. 2022). A key factor limiting gene flow in sheep in general is the limited use of artificial insemination, and other reproductive technologies, due to physiological (e.g. cervical morphology) and financial constraints (Rupp et al. 2016). Substructure within the NSIP Rambouillet, could also be attributed to genetic drift in the relatively small population, possible differences in breeding objectives, and the historic use of non-Rambouillet breeding stock, particularly in Flock 1. Subdivision of flocks is notable in the context of developing an across-flock genetic evaluation program, which benefits from increased connectivity among flocks. Together, these pedigree- and genomic-based evaluations quantify the current levels of diversity and inbreeding found within the NSIP Rambouillet population and provide insight into relationships among flocks.

In contrast to the other NSIP populations evaluated, the Rambouillet were both distinct from and more diverse than the NSIP Suffolk and Katahdin. The NSIP Rambouillet also had the lowest mean inbreeding coefficient, providing further support of their greater diversity compared to the other NSIP breeds. The greater diversity of NSIP Rambouillet compared to Suffolk and Katahdin supports previous results in which these breeds were compared using microsatellites, although both the Rambouillet and Suffolk sheep in that study were European in origin (Blackburn et al. 2011). Another recent SNP-based analysis that included both US Rambouillet and Katahdin sheep also found the Rambouillet to have less inbreeding than the Katahdin (Becker et al. 2024). With regards to NSIP sheep particularly, the calculated effective population size for NSIP Katahdin based on pedigree data ranged from 42.2 to 451 (Nilson et al. 2024) while that based on molecular analysis estimated N_e_ of the NSIP Suffolk population to be 79.5 (Wilson et al. 2022). In this study, the minimum estimated N_e_ for the NSIP Rambouillet of 87 was likely biased downward due to lack of completeness of the pedigree beyond 4 generations. Although the maximum estimate for Rambouillet did not exceed that of the Katahdin, the estimates of N_e_ for the NSIP Rambouillet support that there is sufficient genetic diversity in this population.

The generation interval for the NSIP Rambouillet of 3.4 years is greater than that reported for both the NSIP Suffolk and Katahdin (Wilson et al. 2022; Nilson et al. 2024). This may be due to differences in the production environment among breeds as Rambouillet ewes are generally mated to lamb for the first time at 2 years of age compared to 1 year of age for breeds reared in less arid regions. Additionally, selecting for dual-purpose production may also contribute to the greater overall genetic diversity of the NSIP Rambouillet relative to the other NSIP breeds included in the study, which are primarily selected for growth rate, carcass characteristics, reproductive efficiency, or internal parasite resistance (Wildeus 1997; Notter et al. 2012).

Population structure in the NSIP Rambouillet was in part driven by over half of the samples originating from one flock. As in the analysis with the other NSIP breeds, randomized thinning and pruning of the population were conducted to reduce bias in the analyses due to the overrepresentation of this flock in the sample. Regardless, Flock 1 was still responsible for a large proportion of the sample and ultimately of the US NSIP Rambouillet population. It should be emphasized that a minority of US sheep are enrolled in the NSIP. Therefore, although this study includes a large proportion of the diversity of the NSIP Rambouillet, it is likely that there is additional diversity in Rambouillet sheep in the US that is not represented in NSIP.

Cluster and PC analyses supported that the European Rambouillet sample was intermediate to the NSIP Rambouillet samples; this was demonstrated in PC1, which generally placed European Rambouillet between sheep of Flock 1 and the other NSIP flocks, and in cluster analyses that showed shared ancestry of the European sample most all NSIP flocks. This result suggests either a more prominent founder effect within Flock 1 compared to the others or divergent selection from the founding European individuals. It may also reflect the more prominent incorporation of Merino genetics into Flock 1 although, as visualized in the PC analysis, Flock 1 individuals did not cluster near the Merino samples included in the study. Despite the substructure within the NSIP Rambouillet and relative diversity among the three NSIP samples, the NSIP Rambouillet was least diverse in comparison to international wool breeds.

All international populations evaluated within this study were from flocks sampled in southwestern Europe (Kijas et al. 2012). The geographic barrier between the NSIP Rambouillet and flocks representing the international subset as well an assumed founder effect in the origination of the NSIP population provides sensible explanations for the limited gene flow observed between NSIP and other samples. The use of Merino sires, popular throughout Europe, likely contributed to both the relationships among and the genetic diversity of European breeds such as those included in the study (Kijas et al. 2012; Selli et al. 2021). Additionally, greater diversity in the European sample compared to NSIP breeds is expected simply due to the scale of production; an estimated 57.5 million sheep were present in 2023 in the European Union compared to 5 million estimated in the United States (US Department of Agriculture 2023; Eurostat 2024). For NSIP breeders, however, substructure present in the NSIP Rambouillet suggests there is variation in the population upon which breeders can select, but constrained gene flow among flocks can limit the accuracy and application of genetic evaluation. As the sheep industry moves to incorporate genomics into the genetic evaluation, despite the benefits, there is a risk of greater losses in diversity over time. By assessing the extent of diversity present in the current NSIP flock, we established a benchmark from which to measure change. If appreciable losses are detected, opportunities to implement strategies to minimize further losses in genetic diversity, such as optimal contribution selection (Meuwissen 2009) could be considered. Also, additional diversity could be introduced by actively seeking increased membership in NSIP amongst US Rambouillet producers; the inclusion of additional flocks would almost certainly increase the genetic diversity of the population by integrating genetics that may not be represented in the current sample.

## Supplementary Material

Rambouillet_Supplement_R1_esaf079

## Data Availability

The genotype data are available from the corresponding author upon reasonable request.
