# Supplementary material for: Genetic diversity of US Rambouillet in the National Sheep Improvement Program compared to other sheep breeds
Source: J Hered. 2025 Oct 3;117(2):211–21. doi: 10.1093/jhered/esaf079 (PMC13017815; doi:10.1093/jhered/esaf079)
Supplement: Rambouillet_Supplement_R1_esaf079 [file rambouillet_supplement_r1_esaf079.docx]

Supplementary Figure 1. Principal component (PC) 1 vs PC3 of the NSIP Rambouillet identified by flock (N=500).


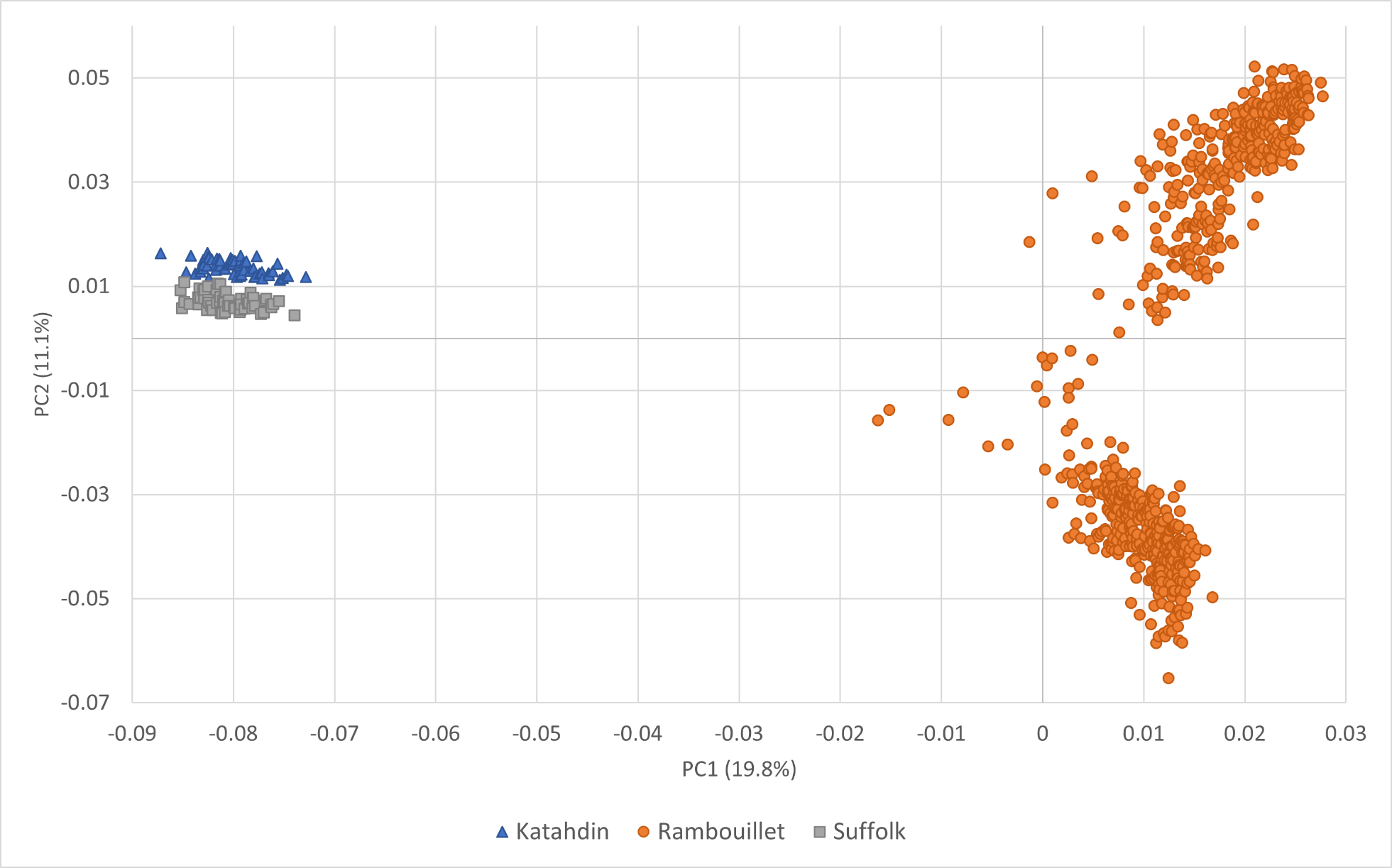


Supplementary Figure 2: Principal component 1 (PC1) vs PC2 of the three NSIP breeds without subsampling. 21,169 SNP loci were included in the analysis.


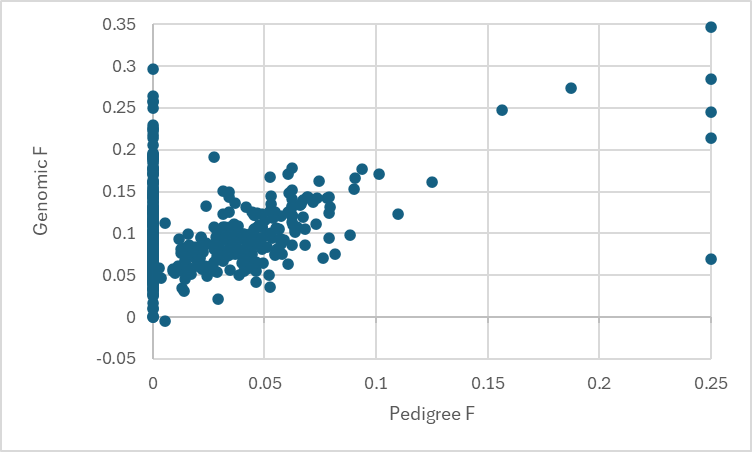


Supplementary Figure 3: Pedigree vs. genomic estimates of inbreeding (F) for NSIP Rambouillet. The correlation between values was 0.340, or 0.72 if sheep with a pedigree-estimated F of zero are removed.

Supplementary Figure 4: Principal component (PC) 1 vs. PC3 of the NSIP Rambouillet and other wool breeds. PC3 serves to separate the Churra further from the Merino samples while the NSIP Rambouillet falls into one large cluster.

Supplementary Table 1: Pairwise F_ST_ for the eight NSIP Rambouillet flocks (one flock removed due to representation by only 1 individual). All comparisons differed significantly except for that between Flocks 3 and 7 (bold).

|  | Flock 1 | Flock 2 | Flock 3 | Flock 4 | Flock 5 | Flock 6 | Flock 7 |
| --- | --- | --- | --- | --- | --- | --- | --- |
| Flock 2 | 0.059 |  |  |  |  |  |  |
| Flock 3 | 0.064 | 0.081 |  |  |  |  |  |
| Flock 4 | 0.100 | 0.121 | 0.110 |  |  |  |  |
| Flock 5 | 0.076 | 0.090 | 0.083 | 0.086 |  |  |  |
| Flock 6 | 0.090 | 0.107 | 0.072 | 0.136 | 0.104 |  |  |
| Flock 7 | 0.064 | 0.081 | **0.001** | 0.108 | 0.081 | 0.072 |  |
| Flock 8 | 0.094 | 0.117 | 0.102 | 0.149 | 0.121 | 0.142 | 0.104 |

Supplementary Table 2: F_IS_ for each breed sample calculated from 21,062 loci. Values in bold are significantly greater than zero.

| Australian Industry Merino | Australian Merino | Australian Poll Merino | Chinese Merino | Churra | Merino Landschaf | Ojalada | NSIP Rambouillet | European Rambouillet | Rasa Aragonesa |
| --- | --- | --- | --- | --- | --- | --- | --- | --- | --- |
| 0.024 | **0.036** | **0.011** | -0.014 | 0.013 | 0.000 | 0.001 | **0.038** | **0.037** | **0.005** |
